# Genetic and demographic recovery of an isolated population of brown bear *Ursus arctos* L., 1758

**DOI:** 10.7717/peerj.1928

**Published:** 2016-04-28

**Authors:** Elena G. Gonzalez, Juan C. Blanco, Fernando Ballesteros, Lourdes Alcaraz, Guillermo Palomero, Ignacio Doadrio

**Affiliations:** 1 Departamento de Biodiversidad y Biología Evolutiva, Museo Nacional de Ciencias Naturales, MNCN-CSIC, Madrid, Spain; 2 Fundación Oso Pardo, Santander, Spain

**Keywords:** Cantabrian brown bear, Recovery, Migration, Gene flow, Conservation, Ursus arctos

## Abstract

The brown bear *Ursus arctos* L., 1758 population of the Cantabrian Mountains (northwestern Spain) became isolated from other bear populations in Europe about 500 years ago and has declined due to hunting and habitat degradation. At the beginning of the 20th century, the Cantabrian population split into eastern and western subpopulations, and genetic exchange between them ceased. In the early 1990s, total population size was estimated to be < 100 bears. Subsequently, reduction in human-caused mortality has brought about an increase in numbers, mainly in the western subpopulation, likely promoting male-mediated migration and gene flow from the western nucleus to the eastern. To evaluate the possible genetic recovery of the small and genetically depauperate eastern subpopulation, in 2013 and 2014 we genotyped hair and faeces samples (116 from the eastern subpopulation and 36 from the western) for 18 microsatellite markers. Data from the annual count of females with cubs of the year (COY) during the past twenty-six years was used to analyze demographic changes. The number of females with COY fell to a minimum of seven in the western and three in eastern subpopulations in the biennium 1993–1994 and reached a respective maximum of 54 and 10 individuals in 2013–2014. We also observed increased bear dispersal and gene flow, mainly from the western to the eastern subpopulation. Of the 26 unique genotypes detected in the eastern subpopulation, 14 (54%) presented an admixture composition, and seven (27%) were determined to be migrants from the western subpopulation. Hence, the two separated and clearly structured subpopulations identified in the past currently show some degree of genetic admixture. This research shows the partial demographic recovery and a change in genetic composition due to migration process in a population of bears that has been isolated for several centuries.

## Introduction

In recent centuries, large carnivore populations have been declining worldwide due to human intervention and habitat destruction (Treves & Karanth, 2003), but in the past 40 years, species resilience, species protection, land sharing programmes, and ongoing conservation of wilderness zones has supported partial recovery in areas of Europe and America (Chapron et al., 2014; Gilroy, Ordiz & Bischof, 2015; Gompper, Belant & Kays, 2015). The brown bear *Ursus arctos* may be a good model for study of the impact of population distribution on the genetic diversity of large mammals (Davison et al., 2011; Karamanlidis et al., 2012; Straka et al., 2012; Taberlet & Bouvet, 1994). Once widespread throughout Europe, most brown bear populations have undergone a reduction in numbers and geographic distribution over the past millennium, particularly since the 15th century, as a result of anthropogenic factors (Taberlet & Bouvet, 1994; Valdiosera et al., 2008).

The recent increase, expansion, and secondary contact processes occurring in some fragmented bear populations may have helped to improve their demographic status. An example of this is the recovery of the brown bear in Finland (Hagen et al., 2015), where the range contraction a century ago produced genetic structuring and led to at least two separate populations. Conservation during the second half of the 20th century, accompanied by immigration from Russia (Kopatz et al., 2014; Kopatz et al., 2012), has resulted in increasing numbers of bears, which dispersed further north and west over time. As a result, the Finnish population increased, and genetic screening has provided evidence of range expansion and gradual disappearance of population substructure along with increasing genetic diversity and admixture. Assignment probabilities of individuals suggested expansion from the southern subpopulation of Finland, which was supported by gradually increasing heterozygosity, allelic richness, and average numbers of alleles in the southern subpopulation (Hagen et al., 2015).

Nevertheless, some populations are so small and fragmented that natural recovery has failed in spite of the costly conservation programmes implemented by governments and NGOs (Woodroffe, 2001). The threshold under which a population is unrecoverable is difficult to assess, depending on a complex mixture of demographic, genetic, ecological, and socio-economic factors that are difficult to quantify and not always well known (Allendorf & Luikart, 2007).

Most brown bear populations assumed to have more than 100 individuals in 1950–1970 are currently recovering (Chapron et al., 2014), but smaller populations that have been isolated and cannot be rescued by large neighbouring populations have faced challenges to recovery or have become extinct. For some of these populations their genetic variability is still unknown. That has been the case with four isolated brown bear populations in Western Europe that survived at least until the 1980s in the Apennines (Italy), the eastern Alps (Italy), the Pyrenees (France and Eastern Spain), and the Cantabrian Mountains (Western Spain) (Chapron et al., 2014). In the Pyrenees and the Alps, bears were in decline during the last decades of the 20th century. When there were single or few bears left, populations were restored by introduction of animals from Slovenia (Clark, Huber & Servheen, 2002; Tosi et al., 2015). In the Apennines, after many decades of protection and conservation programs, 51 bears remain, but the population does not seem to be increasing (Ciucci et al., 2015). Of these four populations, the brown bear of the Cantabrian Mountains is the only isolated population in Western Europe showing a clear trend to natural recovery. This population has been isolated from that geographically nearest, the Pyrenean population, for at least 400 years (Nores & Naves, 1993).

During the first decades of the 20th century, the Cantabrian population split into western and the eastern subpopulations separated by a strip of land of 50–100 km wide with poor habitat quality and an accumulation of structures and roads (García et al., 2007; Nores & Naves, 1993). Bears in the two Cantabrian nuclei declined in number until the mid-1990s. In 1982–1995, the western population comprised 50–60 bears and showed an annual decrease of 4–5% (Wiegand et al., 1998). The eastern subpopulation comprised 20–25 bears in 1990 (Palomero, Fernández & Naves, 1993).

Surveys conducted in the late 1990s and early 2000s found significant genetic differentiation between the western and eastern subpopulations, likely increased by the evolutionary processes of genetic drift and selection since the population split (García-Garitagoitia, Rey & Doadrio, 2006; Pérez et al., 2009; Rey et al., 2000). These works indicated that the eastern Cantabrian subpopulation showed some of the lowest genetic variation among brown bear populations in Europe (Swenson, Taberlet & Bellemain, 2011). As consequence, both subpopulations are considered critically endangered in the Red Book of Spanish mammals (Palomero, 2007). Over the past 50 years, the Cantabrian bears seemed on the path to extinction, similar to the Pyrenean and the Alpine populations. However, this trend has been recently changing. Semi-annual monitoring of the Cantabrian bears, based on the number of females and cubs of the year (COY) (Palomero, 2007), and genetic surveys indicate that both the western and eastern subpopulations have increased since the mid-1990s. Despite some controversy about the reliability of the count of females with COY to determine population trends, the reported annual increase from 1990–2000 was 7.5% and 3.0% for the western and eastern subpopulations, respectively (Palomero, 2007). A genetic census conducted in 2006 estimated Nc is 203 bears in the western subpopulation (CI 95% = 168–260) and Nc is 19 (CI 95% = 12–40) bears in the eastern subpopulation (Pérez et al., 2014).

Connectivity was previously detected between Cantabrian brown bear populations; three males belonging genetically to the western subpopulation were found in the eastern subpopulation, and one male from the eastern subpopulation was found in the western subpopulation (Pérez et al., 2009; Rey et al., 2000). However, only in 2008 were two admixed individuals at the western limits of the eastern subpopulation range identified, indicating genetic flow between subpopulations (Pérez et al., 2010). In the most recent studies of Cantabrian bears (Pérez et al., 2014; Pérez et al., 2010), the majority of genetic samples were collected in 2006, with a few from 2008. The majority of the samples providing reliable genotypes were from the western subpopulation, and little information is available on the eastern subpopulation.

The goal of this study was to assess the demographic and genetic effects of reconnection on the eastern Cantabrian brown bear subpopulation. Our hypothesis was that the eastern subpopulation had experienced population growth and altered genetic composition through movements of individuals and effective genetic transfer of alleles from the western subpopulation. We assessed the eastern subpopulation genetic variation and gene flow, investigated possible movements of individuals from the western to the eastern subpopulation, and evaluated the impact of this migration process on genetic diversity. We employed several methods of determining the level of relatedness among individuals and estimated the effective population size (Ne) of the eastern subpopulation.

Complementary to the genetic data, data concerning females with COY from twenty-six years of field-based monitoring in both subpopulations were used to evaluate demographic changes.

## Materials and Methods

### Population monitoring

Bear population monitoring in the Cantabrian Mountains was carried out from 1989–2014, counting females with COY as described by Palomero et al. (2007). Females with COY are the demographic unit of bear populations commonly used to give the best estimate of the total population size. In European populations, in which female bears usually breed every second year, the total number of bears is generally the number of females with cubs of the past year (or the average of the past two years) multiplied by 8–13, since a healthy population is composed of 8–12% females with cubs (Servheen, 1989; Solberg et al., 2006). To distinguish females with COY from one another, number of cubs, physical features, distance between sightings, and concurrent sightings were considered (Ordiz et al., 2007; Palomero et al., 2007). Although the method has been criticised (Fernández-Gil, Ordiz & Naves, 2010; Mattson, 1997), the small size of the Cantabrian population, the sparsely forested habitats of the Cantabrian range because of human perturbations such as agriculture, and the high level of field coverage by the monitoring team allowed adequate data on females with COY to provide information on population trends and a feasible demographic index (Palomero et al., 2007; Palomero et al., 2010). To analyse temporal changes and estimate the semi-annual rate of change in numbers of females with COY we employed generalized linear modelling (GLM), using Poisson regressions because we have count data, using the statistical package (R Development Core Team, 2008).

### Sampling collection and DNA extraction

**Figure 1:**
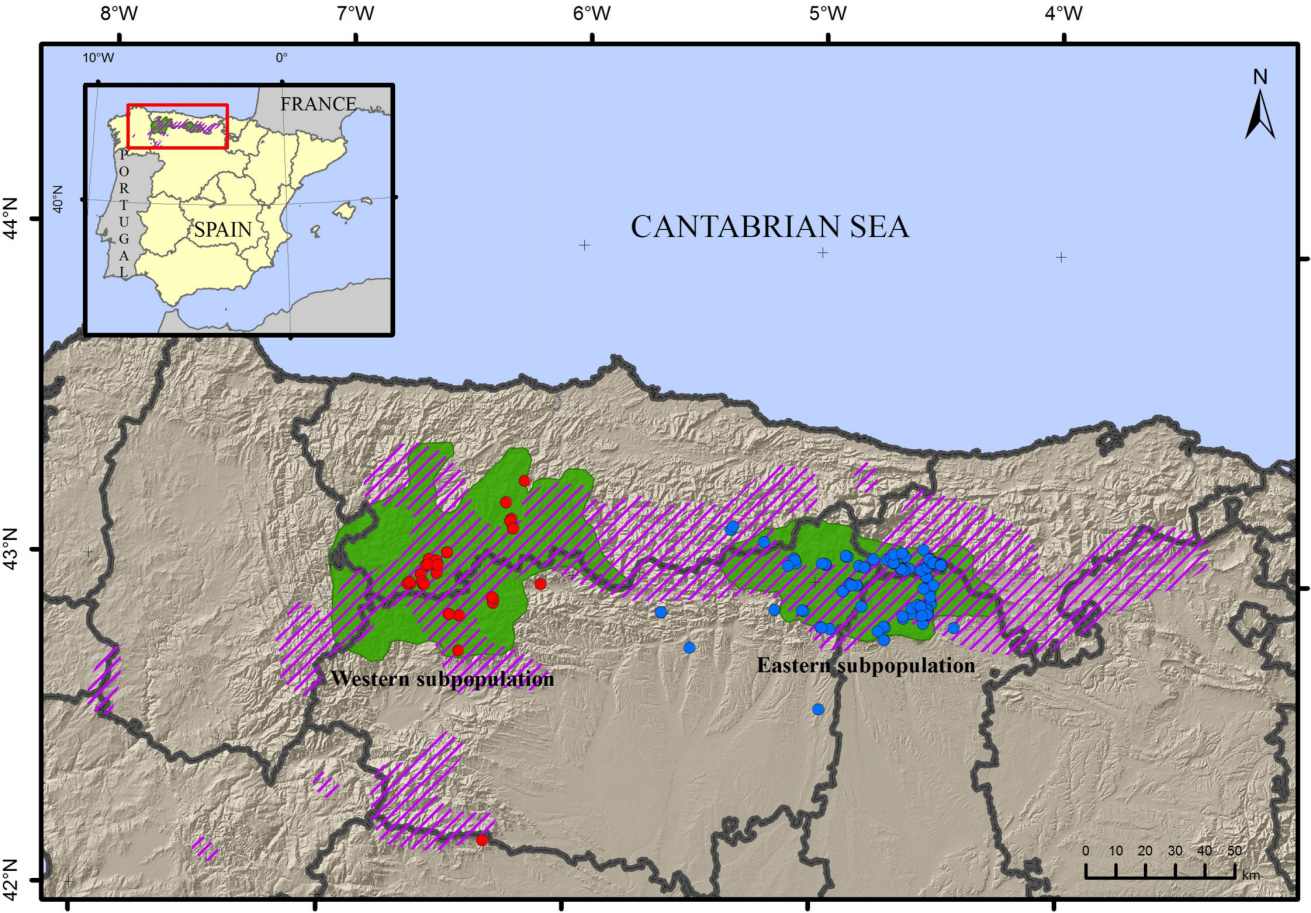
Map of the sampling locations of the brown bear *Ursus arctos*. Samples from the western subpopulation are in blue, samples from the eastern subpopulation are in red. The current distribution area (green) and approximate area of historical occupancy in the 19th century (dashed line) are also indicated.

Non-invasively sampled material from the eastern subpopulation was collected from June 2013–August 2014 in the Cantabrian Mountains (Fig. 1). Similar samples were collected from the western population to compare genetic factors and determine the direction of migration. In total, 152 non-invasive samples including hair (n = 122) and scat (n = 30) were collected. Samples were captured following either systematic (part of the monitoring campaign) or opportunistic (bear-watching and sign surveys or reports of beehive damage from regional rangers) methods, under permission of authorities of the autonomous region of Castile and Leon. The geographic distribution included 116 samples from the eastern subpopulation and 36 from the western (Table S1). Scat samples were dehydrated with silica gel and stored at constant temperature, and hair samples were stored in non-bleached paper envelopes again at constant temperature, until DNA extraction. The research did not involve animal experimentation and complied with international guidelines on ethical behaviour.

DNA was extracted from the hair root using the QIAamp DNA Investigator kit (Qiagen) and from faeces using the QIAamp DNA Stool kit (Qiagen) following manufacturer’s instructions and eluting the DNA in 40 μl of water for hair and 100 μl of water for faeces. The DNA extraction was performed in a room designed for ancient DNA extraction at the Museo Nacional de Ciencias Naturales of Madrid, Spain (MNCN-CSIC), using a tube with no DNA as a negative control for the extraction. No more than 16 samples, including respective negative controls, were processed in one set.

### Microsatellite loci amplification

A set of microsatellite loci specific for *Ursus arctos* (Bellemain & Taberlet, 2004; Taberlet et al., 1997) universally used in other European laboratories for brown bear monitoring and further validated for their sensitivity, species specificity, and performance (Andreassen et al., 2012) were used for our work. The loci (Mu05, Mu09, Mu10, Mu23, Mu50, Mu51, Mu59, Mu61, Mu64, G1A, G1D, G10B, G10C, G10J, G10L, G10O, G10P, G10X) were used following a two-step method for PCR amplification (Taberlet et al., 1997). PCR amplifications consisted of denaturing at 95 °C for 3 min; 40 and 35 cycles (for first and second PCR, respectively) of denaturing at 94 °C for 30 s, annealing for 30 s at 60 °C, and extension at 72 °C for 60 s; followed by 15 min extension at 72 °C. Amplifications were conducted using Qiagen Master Mix (Qiagen) in four PCR multiplexes with six (Mu10, Mu23, Mu50, Mu51, Mu59, and GL10), four (Mu61, G10J, G10O, and G10X), and two with three (Mu64, G1A, and G10C) (Mu05, Mu09, G10B) loci markers. The loci G10P and G1D were amplified separately. In all amplifications a positive and two negative internal controls (one for the extraction and one for the amplification) were included per plate. An individual used as reference for inter-laboratory allele scoring (using DNA extracted from tissue, voucher number MNCN/ADN56456) was included as positive control in all runs. To determine the quality of the DNA extraction and amplification, samples were amplified for all loci, and amplified PCR products were run on an ABI Prism 3730 DNA Analyzer (250–500 LIZ size standard). Allele scoring was performed using GeneMapper v. 3.7 (Applied Biosystems). The locus G10P was found to be monomorphic and was eliminated from further analyses. The samples that showed a reliable genotype for > 1 and ≥ 7 loci were considered positive for the extraction and amplification procedure, respectively. The samples with positive amplification were further amplified at least three additional times for all loci. For creating the consensus genotype dataset from these three repetition per locus, only the genotypes with high reliability (RCI score of ≥ 95%) were used (Miller, Joyce & Waits, 2002). This was performed using the software GIMLET v. 1.3.3 (Valière, 2002). The final dataset used comprised consensus (i.e. unique) genotypes from individuals that presented reliable microsatellite amplification for ≥ 16 and ≥ 14 loci in eastern and western subpopulations, respectively. This threshold criterion for number of loci was imposed in order to increase the discrimination power of the data. For the same reason, a stricter value was applied to the eastern subpopulation by increasing the minimum number of loci for the analyses to 16, given its lower population size and genetic diversity compared to the western subpopulation. Finally, the Probability of Identity [P_ID_, (Paetkau & Strobeck, 1994)], PI for siblings [P_ID-Sib_, (Taberlet & Luikart, 1999)], allelic dropout (ADO), and false allele (FA) values were calculated using the software GIMLET.

Sex was determined by amplification of the genes encoding for the amelogenine proteins AMLX and AMLY, which are specific to ursids (Pagès et al., 2009), and results were confirmed with the amplification of the SRY fragment (Bellemain & Taberlet, 2004; Pagès et al., 2009).

### Genetic diversity analyses

The observed (*H*_O_) and expected (*H*_E_) heterozygosity (Nei, 1978), number of alleles (*N*_A_), and the allelic richness standardized for the smallest sample size (*N*_AR_) (El Mousadik & Petit, 1996) were calculated using the GENEPOP v. 4.0 (Raymond & Rousset, 1995) and FSTAT (Goudet, 2001) programs. Heterozygote deficiency according to departures from Hardy–Weinberg equilibrium, Wright’s *F*_IS_ statistic estimates, and linkage disequilibrium were determined using Markov Chain Monte Carlo (MCMC) runs of 1,000 batches, each of 2,000 iterations, with the first 500 iterations discarded before sampling (Guo & Thompson, 1992). Correction for multiple testing (type I error rates) was performed using the false discovery rate approach (Benjamini & Hochberg, 1995) with the R package QVALUE (Storey, 2002). Samples from each subpopulation were analyzed both independently and combined into a single dataset.

### Genetic and spatial variation between subpopulations

To analyse population differentiation, a Bayesian clustering approach, implemented in the software STRUCTURE (Pritchard, Stephens & Donnelly, 2000), was used. The number of subpopulations (*K*) with the best value of the mean lnProb (D) was calculated assuming an admixed model and a uniform prior probability of *K*. We performed a series of independent runs for *K* of from one to five populations. MCMC consisted of 5 × 10^6^ burn-in iterations followed by 5 × 10^5^ sampled iterations. The modal value of lambda, Δ*K* (Evanno, Regnaut & Goudet, 2005) was also calculated to infer the best value of *K*. Five runs for each value of *K* were conducted to check consistency of results. The output was summarized to correct variance across runs using CLUMMP (Jakobsson & Rosenberg, 2007), and clusters were depicted using DISTRUCT (Rosenberg, 2004) and STRUCTURE HARVESTER (Earl & vonHoldt, 2012). A principal coordinate analysis (PCoA) (Guinand, 1996) was implemented in GENETIX v. 4.05.2 (Belkir et al., 2000) to further validate the genetic clusters obtained with STRUCTURE. The software ARLEQUIN v.3.5 was used to estimate pairwise *F*_ST_-values between the clusters obtained with STRUCTURE.

Finally, we applied a spatial analysis of molecular variance (SAMOVA 1.0, (Dupanloup, Schneider & Excoffier, 2002)) to define partitions of sampling sites that are maximally differentiated from one another without an a priori assumption about population structure. The geographic coordinates for each region indicated the centre of the localities. We tested a range of *K* values from 2–5, using 100 simulated annealing steps.

### Relatedness analyses

The pairwise relatedness (*r*) between individuals in both subpopulations was calculated based on five commonly used estimates of relatedness estimators (COANCESTRY (Wang, 2011)). These included the estimators denoted by LynchLi, LynchRd, QuellerGT, Ritland, and Wang, in Lynch (1988), Lynch & Ritland (1999), Queller & Goodnight (1989), Ritland (1996) and Wang (2002), respectively. We tested 95% confidence intervals for relatedness estimates for all individuals with a reliable genotype against 5000 bootstrap permutations of the data. The mean value for the three types of true relatedness relationships (unrelated individuals, UR, were *r* = 0.0; half-siblings, HS, were *r* = 0.25; and full siblings, FS, were *r* = 0.5) was used as a threshold to classify individuals as UR ≤ 0.25 < HS < 0.5 ≤ FS. Results were presented as the percent of pairs in each classification.

### Estimate of effective population size

Effective population size (Ne) is one of the most important parameters to estimate in small and endangered populations, since it can be used to predict extinction risk and early detection of fragmentation and population decline (Luikart et al., 2010; Skrbinsek et al., 2012). To determine Ne in the eastern subpopulation, we used two approaches that have been shown to be useful for small populations and require only a single distinct genotypic population sample (Skrbinsek et al., 2012). First, we used a method of estimating Ne from linkage disequilibrium (LD) implemented in the software LDNe (Waples & Do, 2008). We calculated estimates assuming random mating and excluded all alleles with frequencies lower (P_crit_) than 0.02, 0.01, and 0.001. Secondly, we implemented an approximate Bayesian computation method to estimate current effective population size (Ne) in ONeSAMP (Tallmon et al., 2008), which can increase accuracy and precision of the previous method (Skrbinsek et al., 2012). Different upper and lower boundaries of the prior distribution were tested to determine the robustness of the results. Given the critical status of the species, we always used a lower boundary of 2 and changed the upper boundary to 50, 200, and 500. Priors of 13–100 were also tested according to the demographic estimates of Ne described in Pérez et al. (2014). In both cases we used a parametric procedure to obtained 95% confidence intervals (CI).

## Results

### Population monitoring

**Figure 2:**
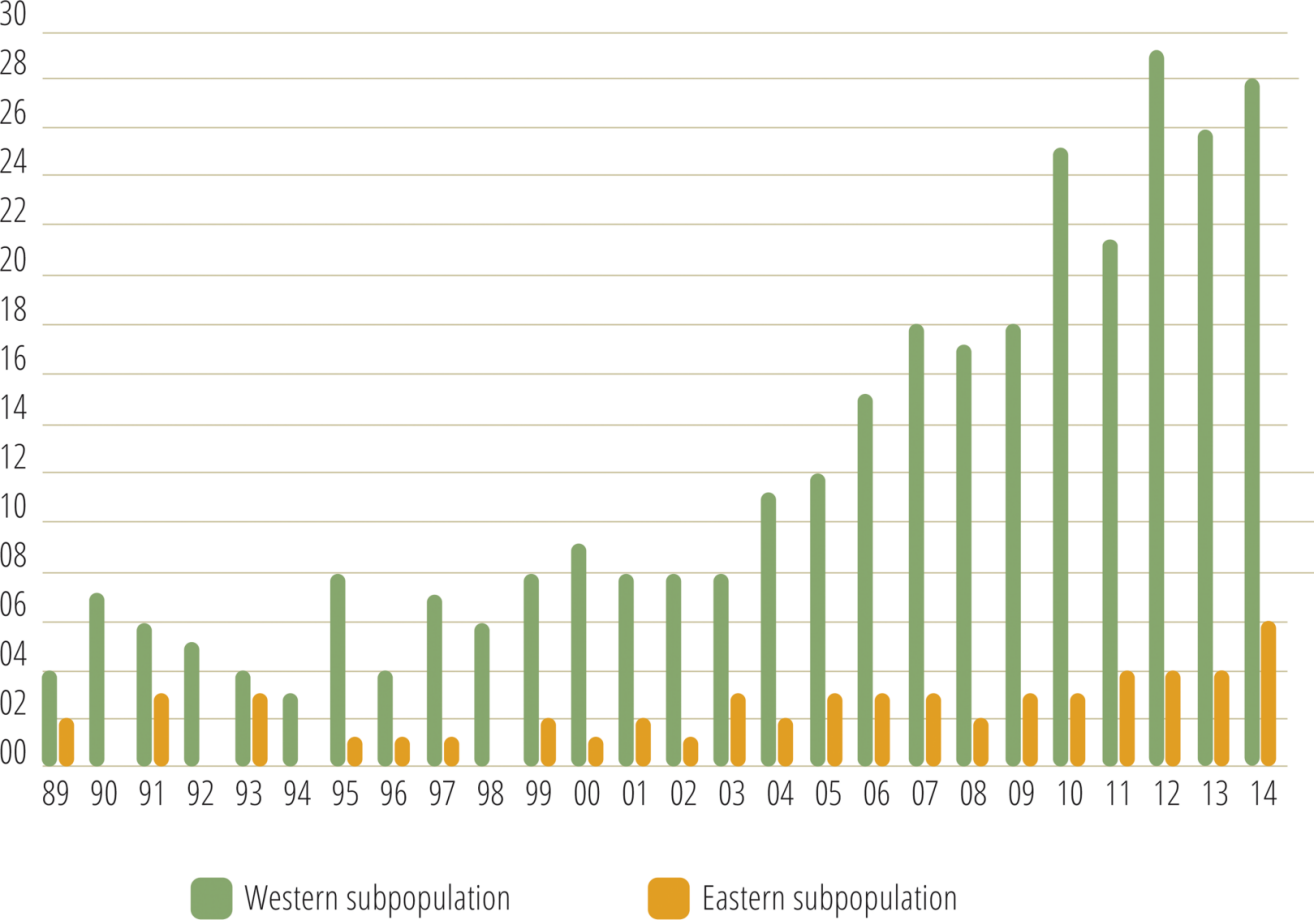
Number of females with COY recorded in the western and eastern brown bear subpopulations of the Cantabrian Mountains from 1989–2014.

**Figure 3:**
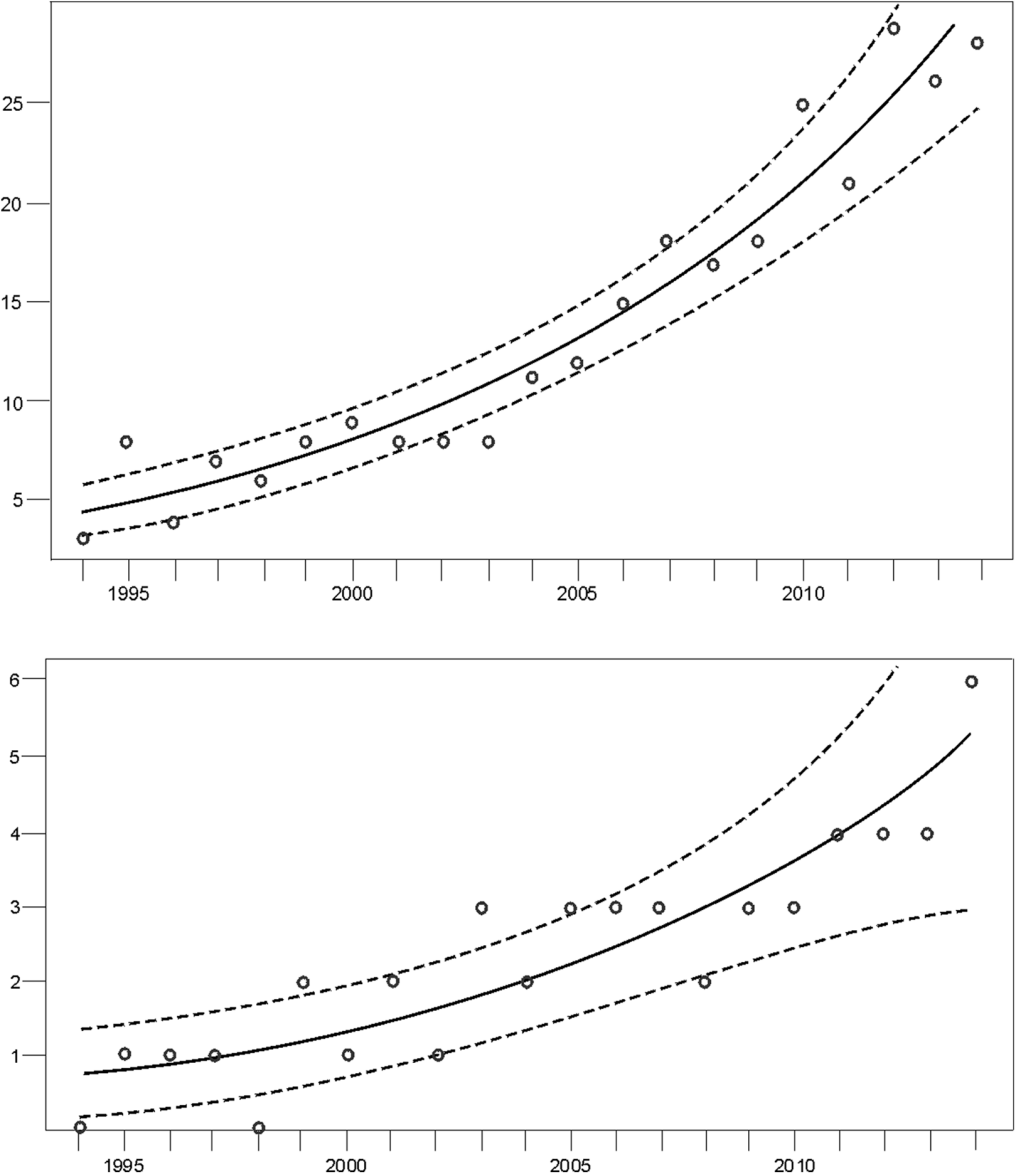
Trend of the number of females with COY (dots) fitted by Poisson regression (lines) from 1994–2014. Data for the (A) western and the (B) eastern subpopulations of brown bears in the Cantabrian Mountains. The 95% confidence limits are indicated with dashed lines.

Monitoring indicated that the Cantabrian bear population has increased steadily from the mid-1990s (Fig. 2). Since the breeding interval of females of this population is normally two years (Palomero et al., 2006), the biennia with minimum and maximum numbers of females with COY found were 1993–1994 and 2013–2014, respectively. In 1993–1994, the number of females with COY was seven in the western and three in the eastern subpopulations. In 2013–2014, 54 and 10 females with COY were recorded in the western and eastern subpopulation, respectively (Fig. 2). Using Poisson regression, the estimated rate of exponential growth from 1994 (when both subpopulations were at the lowest numbers observed in the survey) to 2014 was 10.1% (CI 95%: 7.8–12.4; *p* < 0.0001) for the western subpopulation and 10.4% (CI 95%: 5.0–16.4; *p* = 0.0002) for the eastern subpopulation (Fig. 3).

### Microsatellite dataset preparation and sex determination

A summary of the sample collection and genetic analyses is given in Table S1. Of the 152 samples (n = 122 hair, n = 30 faeces), 144 could be amplified for at least one microsatellite locus, giving an extraction rate of 94.7%. The eight samples that failed amplification were hair samples, 7 of which were from the eastern subpopulation. Of the remaining 144 samples, 90 gave a reliable genotyping profile for ≥ 7 loci, giving an amplification rate of 62.8 % (88.9% for hair and 11.1% for faeces). The final dataset used comprised samples that produced reliable microsatellite genotypes from individuals with unique genotypes for ≥ 16 and ≥ 14 loci in eastern and western subpopulations, respectively. In this way, we obtained 26 unique genotypes in the eastern and 12 in the western subpopulations. As expected, the P_ID_ and P_ID-Sib_ values obtained were low, giving high discriminatory power (mean P_ID_ and mean P_ID-Sib_ 1.35 × 10^−7^ and 4.53 × 10^−5^ for the eastern subpopulation, and 1.37 × 10^−7^ and 3.08 × 10^−4^ for the western subpopulation). The P_ID-Sib_ values obtained with the number of loci used provided higher statistical confidence than the P_ID-Sib_ < 1 × 10^−4^ suggested in distinguishing between full siblings (Waits, Luikart & Taberlet, 2001); hence, we proceeded with the analyses using a minimum number of 16 loci for the eastern subpopulation. Unique genotypes detected in more than one sample were considered to be recapture of an individual and discarded from genetic analyses. This was the case with four samples in the eastern and two in the western subpopulations. Genotyping error results indicated that the majority of errors per locus were due to dropout (11.6%), while the error because of false alleles was 3%.

Sex was determined based on the amplification of the AML and SRY genes in the 144 samples that were positive for amplification. Twenty-nine males and six females were detected in the eastern subpopulation and six males and two females in the western subpopulation. The small number of sexed genotypes and the fact that most of our samples consisted of hair collected on traditional bear marking trees, where there is a significant male bias in scent marking (Clapham et al., 2012), may explain why males are over-represented.

### Genetic diversity

The overall level of genetic diversity based on the number of alleles (mean *N*_A_ and *N*_AR_ of 4.06 and 2.91, respectively) was higher when compared to the values obtained for each subpopulation separately (Table 1). This indicates a high proportion (35.2%) of private alleles in the analysed sample. Five loci, Mu10, Mu50, Mu51, G1A, and Mu05, showed private alleles specific to the eastern subpopulation, whereas only locus G10C showed private alleles for the western subpopulation. The values of the observed and expected diversity in the eastern subpopulation (mean *H*_O_ = 0.541, mean *H*_E_ = 0.530) were similar to those of the western subpopulation (mean *H*_O_ = 0.492, mean *H*_E_ = 0.467) (Table 1) despite the difference in number of individuals analyzed. Both subpopulations showed departure from Hardy–Weinberg equilibrium. However, in the eastern subpopulation, this pattern was chiefly due to significant heterozygote excess (Table 1). Tests for linkage disequilibrium showed a low number of significant pairwise comparisons, which suggests independence of examined loci.

**Table 1:** Summary statistics for each microsatellite locus and each population of *Ursus arctos* (samples collected 2013–2014)*.

	Locus																		
Subpopulation		Mu10	Mu23	Mu50	Mu51	Mu59	G10L	Mu64	G1A	G10C	G10P	Mu61	G10J	G10X	Mu05	Mu09	G1D	G10B	Mean value
Eastern	*N*_A_	5	3	4	3	3	3	4	3	2	6	2	2	5	4	5	2	2	3.41
	*N*_AR_	3.48	2.92	2.48	2.23	2.23	2.23	2.54	2.53	1.99	5.07	2.00	2.00	3.98	3.19	4.17	1.65	2.00	2.75
	*F*_IS_	**0.354**	**0.187**	0.021	0.389	0.473	0.127	**0.734**	0.005	0.412	**−0.093**	**−0.562**	0.006	**0.542**	**0.213**	0.180	0.100	0.405	**0.038**
	*H*_E_	0.602	0.606	0.538	0.465	0.465	0.517	0.493	0.455	0.420	0.795	0.464	0.493	0.704	0.617	0.752	0.124	0.496	0.530
	*H*_O_	0.615	0.618	0.549	0.474	0.474	0.527	0.504	0.464	0.429	0.811	0.473	0.503	0.719	0.629	0.770	0.129	0.507	0.541
Western	*N*_A_	2	3	3	2	3	3	3	2	3	4	2	2	5	2	2	3	2	2.71
	*N*_AR_	1.75	2.89	2.91	2.00	2.55	2.86	2.95	2.00	2.76	3.35	2.00	2.00	4.49	2.00	2.00	2.47	1.95	2.52
	*F*_IS_	0.00	−0,207	0.043	0.431	−0.250	−0.067	0.576	−0.333	−0.414	**−0.618**	−0.636	0.474	0.349	0.437	0.333	0.452	−0.125	**0.026**
	*H*_E_	0.117	0.601	0.500	0.413	0.492	0.500	0.531	0.486	0.517	0.607	0.480	0.483	0.703	0.495	0.444	0.310	0.255	0.467
	*H*_O_	0.125	0.627	0.522	0.431	0.515	0.539	0.567	0.507	0.540	0.636	0.505	0.507	0.750	0.521	0.485	0.325	0.268	0.492
Total	*N*_A_	5	4	4	3	3	4	6	3	3	7	2	2	7	4	6	4	2	4.06
	*N*_AR_	3.33	3.21	2.76	2.15	2.92	2.36	3.34	2.40	2.30	4.77	2.00	2.00	4.14	2.94	4.73	2.20	2.00	2.91

**Note:**

* *N*_A_, number of alleles per locus; *N*_AR_, mean allelic richness standardized to the smallest sample size; mean expected (*H*_E_) and observed (*H*_O_) heterozygosity and mean *F*_IS_, Wright’s statistic per locus and per population. Bold *F*_IS_ values are significant probability estimates after *q*-value correction (*p* < 0.01).

### Population structure and assignment of individuals

**Figure 4:**
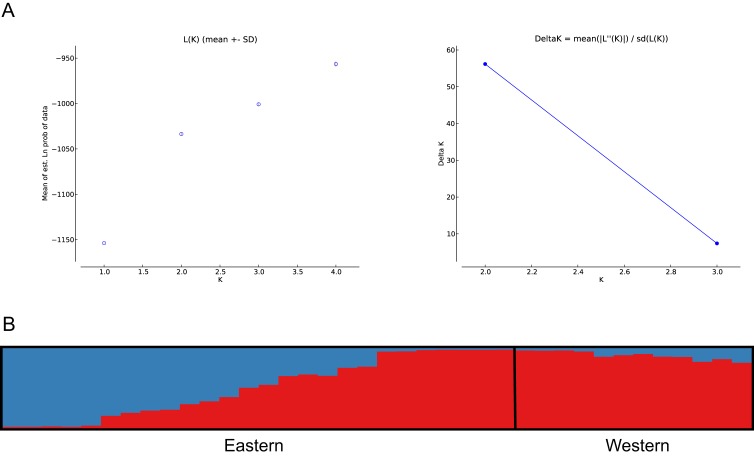
Bayesian clustering analysis based on STRUCTURE. (A) The most likely number of clusters (*K* = 2) detected with the *U. arctos* samples collected in the Cantabrian Mountains expressed as the mean likelihood (log P(D)), and Δ*K*. (B) Representation of the average proportions of memberships (Q) in each of the* K* = 2 inferred clusters. The colours used correspond with the geographic origin of the individuals sampled depicted in Fig. 1.

The number of populations and the assignment of individuals to each population was estimated using Bayesian inference (Pritchard, Stephens & Donnelly, 2000). The analysis showed best population structure, with the highest change in LnP(D), for the model with *K* = 2, corresponding to the subpopulations analysed. Accordingly, the modal value of Δ*K* (Evanno, Regnaut & Goudet, 2005) was shown at *K* = 2 (Fig. 4A). Based on the average proportion of membership (Q) obtained, for the eastern subpopulation, 46% of individuals were assigned to one of the two clusters found (five to the eastern and seven to the western clusters) with a high probability value (Q > 90%), whereas the remaining samples could not be assigned exclusively to a cluster of origin, since Q values ranged from 30–50%. The western subpopulation was clearly defined by a single cluster, with the majority of the samples assigned with high probability (Q > 90%) (Fig. 4B).

**Figure 5:**
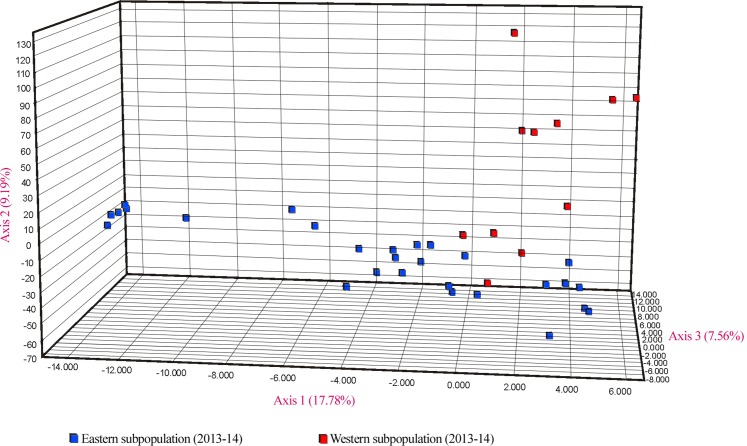
PCoA showing genetic differentiation of the two considered *U. arctos* subpopulations at the Cantabrian Mountains.

Similar levels of genetic differentiation between the two regions were suggested by the significant *F*_ST_ estimate (overall *F*_ST_ = 0.055) and the principal coordinate analysis (PCoA). Results of the PCoA separated samples into the two main groups. Some individuals from the eastern subpopulation overlapped with the western samples, indicating a degree of continuity between the two regions (Fig. 5). However, the three first principal coordinates of the PCoA explained only 34.5% of the molecular variation of the microsatellite loci used, hence this result must be interpreted with caution. Finally, the results of SAMOVA revealed two high *F*_CT_ values for clusters. The highest *F*_CT_ value, 78.77%, corresponded to an arrangement of populations in *K* = 2 clusters. The division into *K* = 3 clusters showed the second highest variance among groups. Both clearly differentiated the western subpopulation as a separate group.

### Relatedness analyses

When we analysed the pairwise relatedness coefficients (*r*) among individuals within each subpopulation, we observed a high proportion of pairs with relatedness values above the half-sib level (> 0.25). The percent of HS and FS pairs ranged from 20.6–45.5% and 16.7–39.4% for the eastern and the western subpopulations, respectively, depending on the coefficient used (Table 2).

**Table 2:** Percentage of pairwise relatedness (*r*) estimates based on three representative genetic relationships found in nature (unrelated individuals, UR, *r* = 0.0; half-siblings, HS, *r* = 0.25; full siblings, FS, *r* = 0.5).

Estimator	LinchLi*			LinchRd			QuellerGT			Ritland			Wang		
Subpopulation	UR	HS	FS	UR	HS	FS	UR	HS	FS	UR	HS	FS	UR	HS	FS
Eastern	54.5	24.0	21.5	78.2	11.7	10.2	68.3	17.5	14.2	79.4	11.7	8.9	56.9	24.9	18.2
Western	60.6	27.3	12.12	72.7	22.7	4.5	77.3	18.2	4.5	83.3	13.6	3.0	62.1	25.8	12.1

**Note:**

* Calculation of *r* was based on relatedness estimators: LynchLi (Lynch, 1988), LynchRd (Lynch & Ritland, 1999), QuellerGT (Queller & Goodnight, 1989), Ritland (Ritland, 1996) and Wang (Wang, 2002) estimators.

### Estimate of effective population size (Ne)

Different values of P_crit_ (the minimum number of allele frequencies) did not alter the Ne values obtained with LDNe. Similarly, the estimate of the effective population size using ONeSAMP for the eastern subpopulation was not sensitive to the prior used, since the values obtained did not vary substantially. Overall, the LDNe software generated higher estimates for Ne (mean 22.4) when compared with the values obtained with ONeSAMP (mean ranging from 12.1–13.7) (Table 3).

**Table 3:** Effective population size (Ne) estimates for the eastern brown bear subpopulation. Values are obtained with the linkage disequilibrium method (implemented in LDNe) and the approximate Bayesian computation method (implemented in ONeSAMP). The lower and upper 95% confidence interval (CI) are also indicated.

LDNe		ONeSAMP	
Priors	Results as mean (95% CI)	Priors	Results as mean (95% CI)
P_crit_ < 0.02	22.4 (20.6–25)	2–50	13.4 (11.5–17.1)
P_crit_ < 0.01	22.4 (20.5–25.1)	2–200	13.7 (11.4–18.5)
P_crit_ < 0.001	22.4 (20.5–25.1)	2–500	12.1 (10.0–16.1)
		13–100	13.1 (11.9–15.3)

## Discussion

The results reveal a steady increase in the number of females with COY for both brown bear subpopulations on the Cantabrian Mountains. Reported migration of some individuals (Pérez et al., 2010) was also confirmed, with the movement of bears mainly from the larger and more densely populated western region to the eastern one. This has had a direct effect on the genetic composition of Cantabrian bears, which has shifted from two separate and clearly structured populations to subpopulations with some admixture. Although showing less genetic diversity and population growth, the Cantabrian bear populations exhibit some similarities to the Finnish brown bears (Hagen et al., 2015) in terms of expansion, connectivity, and homogeneity. In Finland this process resulted in part from immigration of Russian bears (Kopatz et al., 2014; Kopatz et al., 2012), which comprise a large and diverse population. In contrast, the Cantabrian bears have been isolated for several centuries, and nearly a century ago the decreasing population split into two subpopulations. Conservation measures, along with bear resilience, has contributed to increased populations in the two nuclei, and their connection has been partially re-established, although the bear range has seen only limited expansion.

We have attempted to overcome some of the limitations and challenges of non-invasive sampling, including low quality or degraded DNA, by minimizing the genotyping errors and applying robust statistical analyses (Bellemain et al., 2005; Taberlet & Luikart, 1999). To this end, we increased the minimum number of genotyped loci per individual to 16, a higher threshold than the eight (Rey et al., 2000); (Kruckenhauser et al., 2009), 13 (Ciucci et al., 2015), and 14 (Pérez et al., 2014; Pérez et al., 2009) used in previous studies of brown bear genetics, of which we are aware (Table 4). As a consequence, the P_ID_ and P_ID-Sib_ values obtained were low, indicating high discriminatory power. We consider that this difference is the result of an increase in subpopulation size rather than difference in loci used and number of samples analyzed, since the obtained percentage of samples giving a unique genotype was similar among studies.

**Table 4:** Summary of the genetic diversity obtained for the Cantabrian brown bear during the past decade.

Subpopulation	Period of study (years)	No. of loci	No. of genotypes used	*H*_O_	Source
Eastern	1996–1997	≥ 8	20	0.36	Rey et al., 2000
Eastern	1991–1999	≥ 8	27	0.47	García-Garitagoitia, Rey & Doadrio, 2006
Eastern	2006–2008	≥ 14	9	0.25	Pérez et al., 2009
Eastern	2013–2014	≥ 16	26	0.54	This study
Western	2002–2003	≥ 11	91	0.49	García-Garitagoitia, Rey & Doadrio, 2006
Western	2006–2008	≥ 14	31	0.44	Pérez et al., 2009

For the first time, after 26 years of monitoring, we detected changes in the genetic composition in the eastern subpopulation. Previous studies supported the existence of two genetically differentiated subpopulations of brown bear in the Cantabrian Mountains (García-Garitagoitia, Rey & Doadrio, 2006; Pérez et al., 2010; Pérez et al., 2009; Rey et al., 2000), as was found in our study. Hence, the east-west division of the Cantabrian brown bears found previously was confirmed here with the Bayesian assignment analyses of population structure, which defined two clusters corresponding to the subpopulations analysed (García-Garitagoitia, Rey & Doadrio, 2006; Pérez et al., 2010; Pérez et al., 2009; Rey et al., 2000). However, we found larger differences in the genetic composition of the eastern population from that of earlier years, with the presence of individuals showing western population genotypes. In addition we detected a substantial degree of overlap between the two clusters, with a relatively high number of individuals from the eastern subpopulation that could not be unambiguously assigned to their cluster of origin. These individuals were sampled in the eastern region but showed genotypes with ambiguous values of membership coefficient (Q) assignment. Those with low values (Q = 30–50%) were likely the result of admixture, whereas those assigned (Q > 90%) to a different cluster than that of the geographical region in which they were sampled indicate migration between subpopulations, in this case, from the western to the eastern region. Results obtained with *F*_ST_ estimates, PCoA and SAMOVA correspond to the genetic pattern identified by STRUCTURE.

Migration between subpopulations was first detected in 1992 based on genotype composition (Rey et al., 2000), when a male with genetic profile of the western subpopulation was identified on the eastern side. Subsequent migration activity was detected in 2004–2006 with west-east movement of three males (Pérez et al., 2010; Pérez et al., 2009). Gene flow between subpopulations was detected in 2008 based on two genetically admixed individuals sampled in the eastern subpopulation (Pérez et al., 2010). Using Bayesian cluster analysis and sex determination, we observed an increased trend in brown bear dispersion and gene flow between subpopulations. Of the 26 unique genotypes detected in the eastern subpopulation, 14 (54%) presented admixture composition (Q = 30–50%) and seven (27%) were determined to be migrants (Q > 90%) from the western subpopulation. The two migrants successfully sexed were male, an insufficient number to determine whether the migration was a sex-mediated process. However, since dispersal in brown bears has been reported to be sex biased, with males leaving the natal area, while young females establish home ranges close to their mothers (McLellan & Hovey, 2001; Støen et al., 2005), it is likely that this is the case.

The effective population size (Ne) obtained in our study represents the number of individuals that effectively contribute to the population (Frankham, 1995). Our results indicate that Ne varied with the linkage disequilibrium (LD) and Bayesian computation methods, from 22.4 for LDNe to 12.1–13.7 for ONeSAMP. Discrepancies between the two methods are not unexpected, because they rely on different assumptions. The LD method is more restrictive because it assumes selective neutrality, unlinked markers, and a single, closed population (Palstra & Ruzzante, 2008). If migration is taking place among subpopulations, a closed population cannot be assumed, and the results from LDNe could be biased. The estimate obtained with ONeSAMP seems to fit better with our demographic data (COY value in 2014 of 10), since in the eastern subpopulation there are at least ten mature females (females with COY from 2013 plus those from 2014), an unknown number of breeding males, and perhaps some mature females that did not reproduce during the most recent two years. Despite these differences, both methods detected an increased Ne compared to previous estimates (2006, Ne = 9, CI 95% = 8–12; (Pérez et al., 2014)). This can be due to immigrant males in the eastern subpopulation and a consistent increase in the number of reproducing females during recent years. However, our results need further confirmation with larger sample sizes and additional years of sampling. In any case, these numbers are far short of the Ne = 50 adults required to avoid the adverse effects of inbreeding, and the Ne = 500 to avoid extinction due to the inability to cope with environmental change (Frankham, Bradshaw & Brook, 2014). Therefore, management of the eastern brown bear subpopulation should concentrate efforts on enhancing population growth.

The apparent increase of the Cantabrian bear population could be due to the reduction in mortality when effective conservation programs were implemented. For example, the 15-year period 1980–1994 saw 36 cases of illegal killing of bears in the western and 18 cases in the eastern region (Naves et al., 1999), while in 1995–2009, only seven and nine cases, respectively, were reported (Palomero et al., 2011). These nine cases included only one adult female; the others were adult males, young bears, or undetermined. Nevertheless, further cryptic mortality cannot be ruled out, but it is clear that mortality has decreased in recent years due to public awareness and law enforcement. When poaching dropped drastically around the mid-1990s, bear numbers began to increase, filling the gaps in the areas of their subpopulations. Apparently, the progressive saturation of the bear range triggered the dispersal of males. Contrary to the pattern described in Scandinavia (Swenson, Sandegren & So-Derberg, 1998), no dispersal of females has been detected in our study area (Pérez et al., 2010; Pérez et al., 2009; Rey et al., 2000). The migration was more common from west to east, from the subpopulation showing a higher rate of increase to the smaller eastern subpopulation, which entered recovery later. In addition to the increased public tolerance of bears, the rural exodus has led to reversion of the land to a state providing greater coverage and development of processes associated with recovery of natural forest stages (Navarro & Pereira, 2012; Ordiz et al., 2013). The transition from grasslands to shrub and to early-growth oak *Quercus pyrenaica* forests is improving the habitat of the potential corridor between the subpopulations (Naves et al., 2003).

Another factor that may have influenced the direction of the dispersion is conspecific attraction (Stamps, 1988). As rivers run north-south in the Cantabrian range, typical bear movements in past decades were detected in this direction (Clevenger & Purroy, 1991), with bears taking advantage of river corridors to move outside their home area. The connection between the studied subpopulations is hampered by a major highway (AP-66) as well as roads, railways and dams on rivers with a north-south orientation. In recent decades, the permeability of these barriers has not improved; nevertheless, bear presence in the corridor between subpopulations has noticeably increased. In spite of the increased number of bears crossing these barriers, none have been reported killed by traffic, so apparently some have learned to find their way across the passages and tunnels of the highway and through other barriers.

Improved connectivity and increasing population size are presumed to increase the genetic diversity and the long-term viability of populations (Frankham, 1996; Reed et al., 2003). When gene flow is re-established among subpopulations that have been isolated for a long time, spatial population structure decreases, followed by an increase in genetic diversity within subpopulations (Hagen et al., 2015; Ramakrishnan, Musial & Cruzan, 2010). Our results confirmed an increase in genetic flow accompanied by increased genetic diversity. Mean *H*_O_ for the eastern subpopulation (0.54) was similar to that reported for other small brown bear populations, such as the *H*_O_ of 0.50 in Italy in 2011 (n = 45) (Ciucci et al., 2015). Although direct comparison of results among studies presents limitations due to differences in the number of loci and samples used, etc., when compared with the genetic variation of the same subpopulation over the years, we observed an increasing trend in genetic diversity (Table 4). However, interpretation of the results should be made with caution, and a larger number of individuals from the eastern subpopulations should be included in further analyses.

As shown in other studies (Hagen et al., 2015), the migration of bears from the western to the eastern subpopulation has effected a rapid reduction of population substructure and increasing genetic diversity and admixture. Nevertheless, the isolation of the Cantabrian population as a whole prevents the long distance immigration that usually preserves genetic diversity by reshuffling alleles across the landscape (Bialozyt, Ziegenhagen & Petit, 2006; Frankham, 1996). The Cantabrian population has suffered a gradual contraction during the past five centuries (Nores & Naves, 1993), and slow contraction processes have a more pronounced effect on genetic diversity than do rapid contraction processes and also are less likely to preserve the initial genetic diversity; hence leaving the isolated populations with lower genetic difference (Arenas et al., 2012). Severe inbreeding has produced distinct morphological and physical characteristics in brown bears bred in captivity in zoological gardens (Laikre, 1999) as well as in other large carnivores, for example kinked tail, cowlicks, cryptorchidism, and heart defects in the Florida panther *Puma concolor* (Culver, 2010). In spite of the long isolation and the small size of the Cantabrian bear population, especially the eastern subpopulation, no morphological characteristics typical of severe inbreeding have been detected, but further studies are needed.

The demographic monitoring carried out for more than 25 years in the Cantabrian population of *Ursus arctos* has led to increased understanding of changes in a fraction of the bear population. The genetic monitoring programs represent a step forward and could detect demographic and genetic trends and other factors to aid the recovery of this isolated, but seemingly increasing, population.

## Supplemental Information

10.7717/peerj.1928/supp-1 Supplemental Information 1 Unique genotypes for the Cantabrian brown bear samples. Unique genotypes obtained for Cantabrian brown bear individuals collected at the eastern and western subpopulations respectively. The name of the microsatellite loci used is also indicated.

10.7717/peerj.1928/supp-2 Supplemental Information 2 Summary of sample collection and genetic analyses performed on brown bear (*Ursus arctos*) from the Cantabric Mountain.

## Additional Information and Declarations

### Data Deposition

The following information was supplied regarding data availability: Unique genotypes obtained for the Cantabrian brown bear can be found in the Supplementary Information.

## References

[ref-2] Allendorf FW, Luikart G (2007). Conservation and the Genetics of Populations.

[ref-3] Andreassen R, Schregel J, Kopatz A, Tobiassen C, Knappskog PM, Hagen SB, Kleven O, Schneider M, Kojola I, Aspi J, Rykov A, Tirronen KF, Danilov PI, Eiken HG (2012). A forensic DNA profiling system for Northern European brown bears (*Ursus arctos*). Forensic Science International: Genetics.

[ref-4] Arenas M, Ray N, Currat M, Excoffier L (2012). Consequences of range contractions and range shifts on molecular diversity. Molecular Biology and Evolution.

[ref-5] Belkir K, Borsa P, Chickhi L, Raufaste N, Bonhomme F (2000). GENETIX 4.04 Logic el Sous Windows TM, Pour la Génétique des Populations.

[ref-6] Bellemain E, Swenson JE, Tallmon DA, Brunberg S, Taberlet P (2005). Estimating population size of elusive animals with DNA from hunter-collected feces: four methods for brown bears. Conservation Biology.

[ref-7] Bellemain E, Taberlet P (2004). Improved noninvasive genotyping method: application to brown bear (*Ursus arctos*) faeces. Molecular Ecology Notes.

[ref-8] Benjamini Y, Hochberg Y (1995). Controlling the false discovery rate–a practical and powerful approach to multiple testing. Journal of the Royal Statistical Society Series B: Methodological.

[ref-9] Bialozyt R, Ziegenhagen B, Petit RJ (2006). Contrasting effects of long distance seed dispersal on genetic diversity during range expansion. Journal of Evolutionary Biology.

[ref-10] Chapron G, Kaczensky P, Linnell JD, von Arx M, Huber D, Andren H, Lopez-Bao JV, Adamec M, Alvares F, Anders O, Balciauskas L, Balys V, Bedo P, Bego F, Blanco JC, Breitenmoser U, Broseth H, Bufka L, Bunikyte R, Ciucci P, Dutsov A, Engleder T, Fuxjager C, Groff C, Holmala K, Hoxha B, Iliopoulos Y, Ionescu O, Jeremic J, Jerina K, Kluth G, Knauer F, Kojola I, Kos I, Krofel M, Kubala J, Kunovac S, Kusak J, Kutal M, Liberg O, Majic A, Mannil P, Manz R, Marboutin E, Marucco F, Melovski D, Mersini K, Mertzanis Y, Myslajek RW, Nowak S, Odden J, Ozolins J, Palomero G, Paunovic M, Persson J, Potocnik H, Quenette PY, Rauer G, Reinhardt I, Rigg R, Ryser A, Salvatori V, Skrbinsek T, Stojanov A, Swenson JE, Szemethy L, Trajce A, Tsingarska-Sedefcheva E, Vana M, Veeroja R, Wabakken P, Wolfl M, Wolfl S, Zimmermann F, Zlatanova D, Boitani L (2014). Recovery of large carnivores in Europe’s modern human-dominated landscapes. Science.

[ref-11] Ciucci P, Gervasi V, Boitani L, Boulanger J, Paetkau D, Prive R, Tosoni E (2015). Estimating abundance of the remnant Apennine brown bear population using multiple noninvasive genetic data sources. Journal of Mammalogy.

[ref-12] Clapham M, Nevin OT, Ramsey AD, Rosell F (2012). A hypothetico-deductive approach to assessing the social function of chemical signalling in a non-territorial solitary carnivore. PLoS ONE.

[ref-13] Clark JD, Huber D, Servheen C (2002). Bear reintroductions: lessons and challenges. Ursus.

[ref-14] Clevenger AP, Purroy FJ (1991). Ecología del Oso Pardo en España.

[ref-15] Culver M, Negri MHaS (2010). Lessons and insights from evolution, taxonomy and conservation genetics. Ecology and Conservation.

[ref-16] Davison J, Ho SYW, Bray SC, Korsten M, Tammeleht E, Hindrikson M, Østbye K, Østbye E, Lauritzen S-E, Austin J, Cooper A, Saarma U (2011). Late-Quaternary biogeographic scenarios for the brown bear (*Ursus arctos*), a wild mammal model species. Quaternary Science Reviews.

[ref-17] Dupanloup I, Schneider S, Excoffier L (2002). A simulated annealing approach to define the genetic structure of populations. Molecular Ecology.

[ref-18] Earl D, vonHoldt B (2012). STRUCTURE HARVESTER: a website and program for visualizing STRUCTURE output and implementing the Evanno method. Conservation Genetics Resources.

[ref-19] El Mousadik A, Petit RJ (1996). High level of genetic differentiation for allelic richness among populations of the argan tree [*Argania spinosa* (L.) Skeels] endemic to Morocco. Theoretical and Applied Genetics.

[ref-20] Evanno G, Regnaut S, Goudet J (2005). Detecting the number of clusters of individuals using the software STRUCTURE: a simulation study. Molecular Ecology.

[ref-21] Fernández-Gil A, Ordiz A, Naves J (2010). Are Cantabrian brown bears recovering?. Ursus.

[ref-22] Frankham R (1995). Effective population size/adult population size ratios in wildlife: a review. Genetical Research.

[ref-23] Frankham R (1996). Relationship of genetic variation to population size in wildlife. Conservation Biology.

[ref-24] Frankham R, Bradshaw CJA, Brook BW (2014). Genetics in conservation management: revised recommendations for the 50/500 rules, Red List criteria and population viability analyses. Biological Conservation.

[ref-25] García P, Lastra J, Marquínez J, Nores C (2007). Detailed model of shelter areas for the Cantabrian brown bear. Ecological Informatics.

[ref-26] García-Garitagoitia JL, Rey I, Doadrio I, Palomero G, Ballesteros F, Herrero J, Nores C (2006). Variabilidad genética. Demografía, Distribución, Genética y Conservación del Oso Pardo Cantábrico.

[ref-27] Gilroy JJ, Ordiz A, Bischof R (2015). Carnivore coexistence: value the wilderness. Science.

[ref-28] Gompper ME, Belant JL, Kays R (2015). Carnivore coexistence: America’s recovery. Science.

[ref-29] Goudet J (2001). FSTAT, a program to estimate and test gene diversities and fixation indices.

[ref-30] Guinand B (1996). Use of a multivariate model using allele frequency distributions to analyse patterns of genetic differentiation among populations. Biological Journal of the Linnean Society.

[ref-31] Guo SW, Thompson EA (1992). Performing the exact test of Hardy-Weinberg proportion for multiple alleles. Biometrics.

[ref-32] Hagen SB, Kopatz A, Aspi J, Kojola I, Eiken HG (2015). Evidence of rapid change in genetic structure and diversity during range expansion in a recovering large terrestrial carnivore. Proceedings of the Royal Society of London B: Biological Sciences.

[ref-33] Jakobsson M, Rosenberg NA (2007). CLUMPP: a cluster matching and permutation program for dealing with label switching and multimodality in analysis of population structure. Bioinformatics.

[ref-34] Karamanlidis AA, Straka M, Drosopoulou E, de Gabriel Hernando M, Kocijan I, Paule L, Scouras Z (2012). Genetic diversity, structure, and size of an endangered brown bear population threatened by highway construction in the Pindos Mountains, Greece. European Journal of Wildlife Research.

[ref-35] Kopatz A, Eiken HG, Aspi J, Kojola I, Tobiassen C, Tirronen KF, Danilov PI, Hagen SB (2014). Admixture and gene flow from Russia in the recovering Northern European brown bear (*Ursus arctos*). PLoS ONE.

[ref-36] Kopatz A, Eiken HG, Hagen SB, Ruokonen M, Esparza-Salas R, Schregel J, Kojola I, Smith ME, Wartiainen I, Aspholm PE, Wikan S, Rykov AM, Makarova O, Polikarpova N, Tirronen KF, Danilov PI, Aspi J (2012). Connectivity and population subdivision at the fringe of a large brown bear (*Ursus arctos*) population in North Western Europe. Conservation Genetics.

[ref-37] Kruckenhauser L, Rauer G, Däubl B, Haring E (2009). Genetic monitoring of a founder population of brown bears (*Ursus arctos*) in central Austria. Conservation Genetics.

[ref-38] Laikre L (1999). Conservation genetics of Nordic carnivores: lessons from zoos. Hereditas.

[ref-39] Luikart G, Ryman N, Tallmon DA, Schwartz MK, Allendorf FW (2010). Estimation of census and effective population sizes: the increasing usefulness of DNA-based approaches. Conservation Genetics.

[ref-40] Lynch M (1988). Estimation of relatedness by DNA fingerprinting. Molecular Biology and Evolution.

[ref-41] Lynch M, Ritland K (1999). Estimation of pairwise relatedness with molecular markers. Genetics.

[ref-42] Mattson DJ (1997). Sustainable grizzly bear mortality calculated from counts of females with cubs-of-the-year: an evaluation. Biological Conservation.

[ref-43] McLellan BN, Hovey FW (2001). Natal dispersal of grizzly bears. Canadian Journal of Zoology.

[ref-44] Miller CR, Joyce P, Waits LP (2002). Assessing allelic dropout and genotype reliability using maximum likelihood. Genetics.

[ref-45] Navarro LM, Pereira HM (2012). Rewilding abandoned landscapes in Europe. Ecosystems.

[ref-46] Naves J, Wiegand T, Fernández A, Stephan T (1999). Riesgo de Extinción del Oso Pardo Cantábrico.

[ref-47] Naves J, Wiegand T, Revilla E, Delibes M (2003). Endangered species constrained by natural and human factors: the case of brown bears in Northern Spain. Conservation Biology.

[ref-48] Nei M (1978). Estimation of average heterozygosity and genetic distance from a small number of individuals. Genetics.

[ref-49] Nores C, Naves J, Naves J, Palomero G (1993). Distribución histórica del oso pardo en la Península Ibérica. El Oso Pardo (Ursus arctos) en España.

[ref-50] Ordiz A, Rodríguez C, Naves J, Fernández A, Huber D, Kaczensky P, Mertens A, Mertzanis Y, Mustoni A, Palazón S, Quenette PY, Rauer G, Swenson JE (2007). Distance-based criteria to identify minimum number of brown bear females with cubs in Europe. Ursus.

[ref-51] Ordiz A, Støen O-G, Sæbø S, Sahlén V, Pedersen BE, Kindberg J, Swenson JE (2013). Lasting behavioural responses of brown bears to experimental encounters with humans. Journal of Applied Ecology.

[ref-52] Paetkau D, Strobeck C (1994). Microsatellite analysis of genetic variation in black bear populations. Molecular Ecology.

[ref-53] Pagès M, Maudet C, Bellemain E, Taberlet P, Hughes S, Hänni C (2009). A system for sex determination from degraded DNA: a useful tool for palaeogenetics and conservation genetics of ursids. Conservation Genetics.

[ref-94] Palomero G, Ballesteros F, Blanco JC, García-Serrano A, Herrero J, Nores C, Palomero G, Ballesteros F, Herrero J, Nores C (2006). Evolución demográfica y espacial. Demografía, Distribución, Genética y Conservación del Oso Pardo Cantábrico.

[ref-54] Palomero G, Palomo LJ, Gisbert J, Blanco JC (2007). *Ursus arctos* L., 1758. Atlas y Libro Rojo de los Mamíferos Terrestres de España.

[ref-55] Palomero G, Ballesteros F, Nores C, Blanco JC, Herrero J, García-Serrano A (2007). Trends in number and distribution of Brown bear females with cubs-of-the-year in the Cantabrian mountains, Spain. Ursus.

[ref-56] Palomero G, Ballesteros F, Nores C, Blanco JC, Herrero J, García-Serrano A (2010). Are brown bears recovering in the Cantabrian Mountains? Reply to Fernández-Gil et al. Ursus.

[ref-57] Palomero G, Blanco JC, Ballesteros F, García-Serrano A, Herrero J, Nores C (2011). Record de osas con crías en el occidente cantábrico. Quercus.

[ref-58] Palomero G, Fernández A, Naves J, Naves J, Palomero G (1993). Demografía del oso pardo en la Cordillera Cantábrica. El Oso Pardo (Ursus arctos) en España.

[ref-59] Palstra FP, Ruzzante DE (2008). Genetic estimates of contemporary effective population size: what can they tell us about the importance of genetic stochasticity for wild population persistence?. Molecular Ecology.

[ref-60] Pérez T, Naves J, Vázquez JF, Fernández-Gil A, Seijas J, Albornoz J, Revilla E, Delibes M, Domínguez A (2014). Estimating the population size of the endangered Cantabrian brown bear through genetic sampling. Wildlife Biology.

[ref-61] Pérez T, Naves J, Vázquez JF, Seijas J, Corao A, Albornoz J, Domínguez A (2010). Evidence for improved connectivity between Cantabrian brown bear subpopulations. Ursus.

[ref-62] Pérez T, Vázquez F, Naves J, Fernández A, Corao A, Albornoz J, Domínguez A (2009). Non-invasive genetic study of the endangered Cantabrian brown bear (*Ursus arctos*). Conservation Genetics.

[ref-63] Pritchard JK, Stephens M, Donnelly P (2000). Inference of population structure using multilocus genotype data. Genetics.

[ref-64] Queller DC, Goodnight KF (1989). Estimating relatedness using molecular markers. Evolution.

[ref-1] R Development Core Team (2008). R: A Language and Environment for Statistical Computing.

[ref-65] Ramakrishnan AP, Musial T, Cruzan MB (2010). Shifting dispersal modes at an expanding species’ range margin. Molecular Ecology.

[ref-66] Raymond M, Rousset F (1995). GENEPOP 3.3: population genetic software for exact test and ecumenism. Journal of Heredity.

[ref-67] Reed DH, O’Grady JJ, Brook BW, Ballou JD, Frankham R (2003). Estimates of minimum viable population sizes for vertebrates and factors influencing those estimates. Biological Conservation.

[ref-68] Rey I, Doadrio I, Palomero G, Taberlet P, Waits L, Layna JF, Heredia B, Palomero G, Doadrio I (2000). Individualización, determinación del sexo y variabilidad genética del núcleo oriental de oso pardo de la Cordillera Cantábrica. La Conservación del Oso Pardo en Europa: un Reto de Cara al Siglo XXI.

[ref-69] Ritland K (1996). Estimators for pairwise relatedness and inbreeding coefficients. Genetics Research.

[ref-70] Rosenberg NA (2004). Distruct: a program for the graphical display of population structure. Molecular Ecology Notes.

[ref-71] Servheen C (1989). Monitoring of bear populations. Environmental Encounters Series, Council of Europe.

[ref-72] Skrbinsek T, Jelencic M, Waits L, Kos I, Jerina K, Trontelj P (2012). Monitoring the effective population size of a brown bear (*Ursus arctos*) population using new single-sample approaches. Molecular Ecology.

[ref-73] Solberg KH, Bellemain E, Drageset O-M, Taberlet P, Swenson JE (2006). An evaluation of field and non-invasive genetic methods to estimate brown bear (*Ursus arctos*) population size. Biological Conservation.

[ref-74] Stamps JA (1988). Conspecific attraction and aggregation in territorial species. American Naturalist.

[ref-75] Støen O-G, Bellemain E, Sæbø S, Swenson JE (2005). Kin-related spatial structure in brown bears *Ursus arctos*. Behavioral Ecology and Sociobiology.

[ref-76] Storey JD (2002). A direct approach to false discovery rates. Journal of the Royal Statistical Society Series B: Statistical Methodology.

[ref-77] Straka M, Paule L, Ionescu O, Štofík J, Adamec M (2012). Microsatellite diversity and structure of Carpathian brown bears (*Ursus arctos*): consequences of human caused fragmentation. Conservation Genetics.

[ref-78] Swenson JE, Sandegren F, So-Derberg A (1998). Geographic expansion of an increasing brown bear population: evidence for presaturation dispersal. Journal of Animal Ecology.

[ref-79] Swenson JE, Taberlet P, Bellemain E (2011). Genetics and conservation of European brown bears Ursus arctos. Mammal Review.

[ref-80] Taberlet P, Bouvet J (1994). Mitochondrial DNA polymorphism, phylogeography, and conservation genetics of the brown bear *Ursus arctos* in Europe. Proceedings of the Royal Society of London B: Biological Sciences.

[ref-81] Taberlet P, Camarra JJ, Griffin S, Uhres E, Hanotte O, Waits LP, Dubois-Paganon C, Burke T, Bouvet J (1997). Noninvasive genetic tracking of the endangered Pyrenean brown bear population. Molecular Ecology.

[ref-82] Taberlet P, Luikart G (1999). Non-invasive genetic sampling and individual identification. Biological Journal of the Linnean Society.

[ref-83] Tallmon DA, Koyuk A, Luikart G, Beaumont MA (2008). ONeSAMP: a program to estimate effective population size using approximate Bayesian computation. Molecular Ecology Resources.

[ref-84] Tosi G, Chirichella R, Zibordi F, Mustoni A, Giovannini R, Groff C, Zanin M, Apollonio M (2015). Brown bear reintroduction in the Southern Alps: to what extent are expectations being met?. Journal for Nature Conservation.

[ref-85] Treves A, Karanth KU (2003). Human-carnivore conflict and perspectives on carnivore management worldwide. Conservation Biology.

[ref-86] Valdiosera CE, Garcia-Garitagoitia JL, Garcia N, Doadrio I, Thomas MG, Hanni C, Arsuaga JL, Barnes I, Hofreiter M, Orlando L, Gotherstrom A (2008). Surprising migration and population size dynamics in ancient Iberian brown bears (*Ursus arctos*). Proceedings of the National Academy of Sciences.

[ref-87] Valière N (2002). Gimlet: a computer program for analysing genetic individual identification data. Molecular Ecology Notes.

[ref-88] Waits LP, Luikart G, Taberlet P (2001). Estimating the probability of identity among genotypes in natural populations: cautions and guidelines. Molecular Ecology.

[ref-89] Wang JL (2002). An estimator for pairwise relatedness using molecular markers. Genetics.

[ref-90] Wang JL (2011). COANCESTRY: a program for simulating, estimating and analysing relatedness and inbreeding coefficients. Molecular Ecology Resources.

[ref-91] Waples RS, Do C (2008). lDNe: a program for estimating effective population size from data on linkage disequilibrium. Molecular Ecology Resources.

[ref-92] Wiegand T, Naves J, Stephan T, Fernandez A (1998). Assesing the risk of extinction for the brown bear (Ursus arctos) in the Cordillera Cantábrica, Spain. Ecological Monograph.

[ref-93] Woodroffe R, Gittleman JLFSM, Macdonald DW, Wayne RK (2001). Strategies for carnivore conservation: lessons from contemporary extinctions. Carnivore Conservation.

