# Supplementary material for: Genetic and demographic recovery of an isolated population of brown bear Ursus arctos L., 1758
Source: PeerJ. 2016 Apr 28;4:e1928. doi: 10.7717/peerj.1928 (PMC4860320; doi:10.7717/peerj.1928)
Supplement: Supplemental Information 2 [file peerj-04-1928-s002.docx]

| **Table S1** Summary of sample collection and genetic analyses performed on brown bear (*Ursus arctos*) from the Cantabrian Mountain | | | |
| --- | --- | --- | --- |
|  | Subopulation | |  |
|  | Eastern | Western | Total |
| No. of samples | 116 | 36 | 152 |
| No. of faece samples | 11 | 19 | 30 |
| No. of hair samples | 105 | 17 | 122 |
| Possitive samples for amplification* | 70 | 20 | 90 |
| Accepted genotypes** | 26 | 12 | 38 |
| No. of males succesfully sexed | 29 | 6 | 35 |
| No. of females succesfully sexed | 6 | 2 | 8 |
| * Samples with genotypes amplified for ≥ 7 loci | | | |
| ** Samples with genotypes amplified for ≥16 and ≥14 loci in eastern and western subpopulations, respectively used for the analyses. | | | |
