# Supplementary material for: Genetic and demographic recovery of an isolated population of brown bear Ursus arctos L., 1758
Source: PeerJ. 2016 Apr 28;4:e1928. doi: 10.7717/peerj.1928 (PMC4860320; doi:10.7717/peerj.1928)
Supplement: Supplementary file 2 [file peerj-04-1928-s001.docx]

Unique genotypes obtained for Cantabrian brown bear individuals collected at the eastern and western subpopulations respectively.

Population	Microsatellite loci																																	
Eastern	MU10		MU23		MU50		MU51		MU59		G10L		MU64		G1A		G10C		G10P		MU61		G10J		G10X		MU05		MU09		G1D		G10B	
60c	113	113	144	154	99	99	120	122	93	93	159	159	186	186	111	117	104	104	123	123	141	143	58	66	129	131	127	131	137	139	171	171	130	130
24c	?	?	154	154	99	99	122	124	93	93	159	159	186	186	111	117	104	104	141	143	141	143	58	66	129	131	129	143	137	139	?	?	130	130
70c	113	113	144	146	97	99	122	124	93	93	159	159	186	186	111	117	104	104	141	143	141	143	58	66	129	129	129	131	137	139	179	179	130	130
85c	113	123	154	154	99	99	124	124	93	93	155	159	186	186	117	117	104	104	141	143	141	143	58	58	131	131	143	143	137	139	179	179	130	130
13/027c	121	123	154	154	99	99	124	124	93	93	159	159	186	186	115	117	102	104	141	143	143	143	58	58	131	131	143	143	137	139	?	?	?	?
13/013c	113	113	144	154	95	99	122	124	93	95	159	159	?	?	115	117	102	104	123	125	141	143	58	58	129	129	127	129	137	137	?	?	130	130
69c	113	113	144	154	?	?	122	124	93	97	159	159	180	180	117	117	102	104	141	143	141	143	58	58	131	131	127	143	137	145	179	179	128	130
94c	113	113	144	146	95	99	122	124	93	97	157	159	178	196	117	117	104	104	127	143	141	143	58	66	129	129	?	?	137	139	179	179	?	?
74c	113	127	144	144	95	99	122	124	93	97	157	159	?	?	117	117	102	104	143	145	141	143	58	66	129	129	127	143	139	145	179	179	130	130
77c	113	113	144	144	95	99	122	124	93	97	157	159	180	186	117	117	102	104	143	145	141	143	58	66	129	129	127	143	?	?	179	179	128	130
72c	113	127	144	144	95	99	122	124	93	97	157	159	?	?	117	117	102	104	143	145	143	143	58	66	129	129	127	143	139	145	179	179	128	130
81c	127	127	144	146	99	99	122	124	93	97	157	157	186	186	115	115	104	104	125	127	141	143	58	66	127	131	129	129	?	?	179	179	130	130
28c	125	127	144	146	95	99	122	124	93	97	157	159	?	?	117	117	104	104	125	125	141	143	58	58	129	129	127	143	?	?	179	179	128	130
52c	113	113	144	146	95	99	122	124	93	97	157	159	180	186	115	117	104	104	141	143	141	143	66	66	?	?	127	127	119	137	179	179	128	128
18c	113	113	144	146	95	99	122	124	93	93	157	159	180	180	117	117	?	?	125	127	141	143	58	66	119	131	127	127	135	137	179	179	?	?
30c	125	127	144	146	95	99	122	124	93	97	157	159	180	180	117	117	102	104	125	127	143	143	58	66	129	129	127	143	139	145	?	?	128	130
29c	125	127	144	146	95	99	122	124	93	97	157	159	180	180	117	117	104	104	125	127	141	143	58	66	129	129	127	143	?	?	?	?	128	130
45c	127	127	144	146	95	99	122	124	93	97	157	159	180	180	117	117	102	104	127	143	141	143	58	66	123	127	127	143	137	145	?	?	128	128
66c	113	113	144	146	95	99	124	124	93	93	157	159	180	180	117	117	102	104	125	127	141	143	58	66	123	127	127	143	119	139	179	179	128	130
33c	127	127	144	144	95	95	124	124	93	97	157	157	180	180	115	115	104	104	123	127	143	143	66	66	123	127	127	127	145	145	?	?	128	128
91c	113	113	144	146	95	95	124	124	93	97	157	157	180	180	115	117	102	104	127	145	143	143	66	66	119	127	127	143	?	?	179	179	128	128
13/006c	113	113	144	146	95	95	124	124	93	97	157	157	180	180	115	117	102	104	125	125	141	143	66	66	?	?	127	143	119	145	?	?	128	128
13/017c	113	113	144	146	95	95	124	124	93	97	157	157	180	180	115	117	102	104	125	127	143	143	66	66	127	127	127	143	119	145	?	?	128	128
13/009c	113	125	144	146	93	95	124	124	93	93	157	157	180	180	115	117	102	104	125	127	141	143	66	66	127	127	127	143	119	145	179	179	128	128
26c	125	127	144	146	95	95	122	124	93	97	157	157	180	180	115	117	102	104	125	127	143	143	66	66	127	127	127	143	119	145	?	?	128	128
27c	125	127	144	146	95	95	124	124	93	97	157	157	180	180	115	117	102	104	125	127	141	143	66	66	127	127	127	143	119	145	?	?	128	128
Western																																		
9c	?	?	146	146	95	95	124	124	95	95	153	159	180	180	115	117	100	102	125	127	?	?	?	?	?	?	127	127	119	141	179	179	128	128
11c	113	113	144	146	95	95	122	122	95	95	?	?	176	176	117	117	100	102	125	127	?	?	66	66	?	?	143	143	119	119	179	179	128	128
107c	113	113	144	146	95	95	122	124	95	95	?	?	?	?	117	117	102	102	125	127	141	143	66	66	127	127	143	143	?	?	179	179	128	128
4c	113	113	146	152	95	95	124	124	93	95	?	?	?	?	115	117	102	102	125	127	143	143	66	66	129	129	143	143	119	119	?	?	128	128
1c	113	113	146	146	93	93	122	122	93	95	157	159	182	182	117	117	102	104	?	?	141	143	58	58	?	?	127	127	141	141	179	179	128	128
101c	113	113	144	146	95	95	124	124	93	95	157	157	?	?	115	117	102	104	127	143	143	143	66	66	127	127	127	143	?	?	179	179	128	130
2c	?	?	144	152	93	95	124	124	?	?	157	159	180	182	115	117	102	104	125	127	141	143	58	58	127	129	127	127	119	119	173	173	?	?
118c	?	?	144	152	95	99	122	124	93	95	?	?	180	182	115	115	102	102	127	143	141	143	58	66	?	?	143	143	119	141	179	179	128	128
102c	113	121	144	144	93	95	124	124	95	95	?	?	180	180	115	117	102	104	127	129	141	143	58	58	127	131	?	?	?	?	173	179	128	128
106c	?	?	144	146	95	99	122	124	93	95	157	159	?	?	115	117	102	104	125	127	141	143	66	66	125	127	?	?	?	?	179	179	128	130
104c	113	113	144	146	95	99	124	124	93	97	157	157	180	180	115	117	102	104	125	127	141	143	58	66	121	131	127	143	?	?	161	179	?	?
103c	113	113	144	146	95	99	124	124	93	95	157	157	180	180	115	117	102	104	125	127	141	143	58	66	131	131	127	143	?	?	179	179	128	130
